# Demodulation-Oriented Neural Denoising for Long-Sequence I/Q Wireless Signals in Data-Link Receiver Chains

**DOI:** 10.3390/s26144406

**Published:** 2026-07-11

**Authors:** Mingdi Li, Yanbin Li, Qi Feng

**Affiliations:** 1 The 54th Research Institute of China Electronics Technology Group Corporation (CETC54), Shijiazhuang 050011, Chinafq_learning@outlook.com (Q.F.); 2 School of Information and Software Engineering, University of Electronic Science and Technology of China (UESTC), Chengdu 611731, China

**Keywords:** wireless communication, demodulation-oriented denoising, receiver preprocessing, bit error rate, long-sequence I/Q signal, data-link receiver, neural denoising

## Abstract

Reliable demodulation of low-signal-to-noise ratio (SNR) data-link signals is challenging when thermal noise, multipath fading, and structured interference overlap with the target waveform in time and frequency. This paper studies neural denoising as a front-end module in a receiver chain for long-sequence in-phase/quadrature (I/Q) wireless signals. A residual one-dimensional denoising autoencoder (DAE) is inserted before a conventional demodulator to recover the interference-free waveform from corrupted inputs. The denoised outputs are assessed using the waveform-level mean squared error (MSE), output SNR, and demodulated bit error rate (BER). The relative bit error rate (RBER) quantifies the residual BER penalty after denoising within the receiver transition region. Extensive experiments across three practical communication-signal classes and various disturbance/channel-effect conditions demonstrate that neural preprocessing significantly improves low-SNR demodulation accuracy. Crucially, the results show that traditional waveform metrics do not fully reflect receiver-level bit recovery. Compared with convolutional, attention-based, adversarial, and pooling-based baselines, the proposed receiver front end provides lower RBER in the operating region where demodulation is most sensitive to waveform distortion. The results support demodulation-oriented evaluation for neural receiver preprocessing and show that waveform restoration should be verified through the downstream bit-recovery task.

## 1. Introduction

Wireless receivers increasingly operate in dense and time-varying electromagnetic environments. Thermal noise, receiver impairments, multipath propagation, co-channel emissions, and intentional interference can overlap with the target waveform in time and frequency. For broadband or multilayer modulated signals, this overlap reduces the signal-to-noise ratio (SNR) margin available for synchronization, carrier or frequency tracking, symbol-decision operations, and channel decoding. This difficulty is most pronounced in low-SNR scenarios, where minor waveform distortions can trigger a severe bit error rate (BER) penalty after demodulation.

Classical filtering remains useful when the disturbance occupies a separable band or follows a stable statistical model. Practical data-link signals often violate these conditions. Fixed filters may remove useful spectral components, decomposition methods may disrupt phase continuity, and sparse priors may fail when structured interference resembles the target signal. Learning-based denoising offers a data-driven alternative. Receiver deployment, however, requires a stricter criterion than waveform reconstruction. The denoised signal must improve synchronization, symbol decisions, and bit recovery.

This study examines long-sequence complex wireless signals represented by two-channel in-phase/quadrature (I/Q) samples. The primary signal class is a multi-tone DQPSK-FM composite data-link waveform, where DQPSK phase terms are embedded within multiple FM-related frequency components. Two additional datasets, a binary FSK waveform and a frequency-hopping minimum-shift keying (MSK) waveform, are used for generalization tests. The denoiser is inserted before the existing demodulator, and performance is judged at both the waveform level and the receiver output. The architecture is a residual one-dimensional denoising autoencoder (DAE) implemented as a convolutional encoder–decoder without pooling or self-attention. This design prioritizes sample-level phase continuity and symbol-boundary preservation for 8192-point receiver sequences while avoiding the quadratic computational cost associated with full attention mechanisms.

The main contributions are summarized below.

1. A neural denoising front end is integrated into a receiver-side processing chain for long I/Q data-link signals. This study evaluates the complete path from corrupted waveform to demodulated bits, aligning more closely with practical receiver assessment than isolated waveform reconstruction.
2. A residual DAE configuration is analyzed for long I/Q sequences. Pooling and attention are tested as explicit design alternatives so that phase-continuity preservation, symbol-boundary sensitivity, and sequence-length cost can be assessed under the same demodulation chain.
3. A demodulation-oriented evaluation protocol is established. Mean squared error (MSE) and output SNR are used as supporting waveform diagnostics, and relative bit error rate (RBER) measures the residual BER penalty after denoising.
4. Experiments on three real wireless signal classes, representative receiver-side disturbance/channel-effect conditions, and a wide SNR range show that neural preprocessing improves low-SNR demodulation accuracy when the final receiver output is used as the main criterion.

## 2. Related Work

### 2.1. Model-Driven Signal Denoising

Model-driven denoising remains the first reference point for communication-signal restoration. Transform-domain collaborative filtering separates repeated local structures in an overcomplete representation and is highly effective for image denoising [1]. Sparse dictionary learning estimates a compact representation from data and suppresses components that cannot be explained by the learned atoms [2]. Variational mode decomposition separates a signal into band-limited modes and has been widely adopted in nonstationary signal analysis [3]. These methods provide interpretable priors, yet their success depends on a separability assumption. When a communication waveform and interference occupy the same band, a denoiser that removes high-energy disturbance can also damage phase continuity, preamble structure, or symbol transitions.

Radio machine-learning datasets have helped standardize modulation-recognition research, but many of them are generated from short synthetic snippets with simplified channel models [4]. Physical-layer deep learning further showed that raw I/Q samples can support end-to-end learning tasks when the training distribution is well controlled [5]. The present study differs by using long receiver sequences and evaluating denoising through a downstream demodulator. In this setting, waveform smoothness is useful only when it improves synchronization, symbol decisions, and bit recovery.

### 2.2. Learning-Based Denoising for Communication and Sensing Signals

Residual convolutional networks are a natural choice for denoising because they can learn a correction term and preserve low-level signal content. The DnCNN study established residual learning as a strong baseline for Gaussian image denoising [6]. Analysis of sparse signal denoising further shows that convolutional denoisers can learn structured smoothing priors from data [7]. One-dimensional residual convolutional autoencoders have also been used for vibration-signal feature learning, showing that residual paths help sequence models retain details after downsampling [8]. For modulation signals, a dual-residual denoising autoencoder (DAE) with channel attention improved low-SNR signal quality and BER-related outcomes on simulated modulation data [9]. A hybrid long short-term memory (LSTM)-residual network (ResNet) has been applied to V-band receiver noise reduction, combining temporal modeling with residual feature extraction [10]. LSTM autoencoders have also been used for real-time radio technology and modulation classification tasks [11]. These studies support residual and recurrent denoising. Long practical receiver waveforms with a full demodulation chain remain less explored.

Attention mechanisms and transformer-style modules are attractive because they can model long-range dependencies. A transformer-based denoising network has been used to improve angle-of-arrival estimation under non-line-of-sight conditions [12]. Transformer autoencoders have also appeared in high-dimensional sensing tasks such as hyperspectral anomaly detection [13]. Signal-to-image transformation provides another route, as shown by a deep network for transient electromagnetic denoising [14]. Attention models provide global context modeling at the cost of memory. For an 8192-point two-channel waveform, self-attention introduces a quadratic sequence-length term. The receiver mainly requires local waveform continuity around symbols, preambles, and frequency transitions.

Recent work also explores denoising with weaker supervision or generative priors. Subsequence splitting and blind-spot training reduce dependence on paired interference-free targets for general signal denoising [15]. Contrastive denoising autoencoders improve robustness by shaping representations in addition to minimizing pointwise error [16]. DenoMAE uses masked autoencoding ideas for modulation-signal denoising and classification, showing the value of self-supervised pretraining for low-data radio tasks [17]. Diffusion models have been adapted for wireless semantic channels and can provide strong restoration under learned channel distributions [18]. In seismic denoising, diffusion-based unsupervised modeling has also shown promise for complex non-Gaussian disturbance [19]. These methods are important alternatives, although their iterative inference or pretraining requirements may complicate deployment in receiver-side processing.

### 2.3. Evaluation Gap

A recurring weakness in signal-denoising papers is the mismatch between the training objective and the communication objective. MSE and SNR are repeatable waveform metrics, but they cannot capture all synchronization and decision errors. Channel-estimation work has shown that denoising is most valuable when downstream communication performance improves [20]. Massive multiple-input multiple-output (MIMO) channel prediction with meta-learning and deep denoising also emphasizes communication performance under limited data [21]. In the present work, the receiver is available, so BER can be measured directly after denoising. Related receiver-learning and acquisition studies provide adjacent context for burst demodulation and preamble-free timing recovery [22,23,24]. The experimental setup therefore reports the baseline implementation details together with the evaluation protocol.

## 3. System Model and Problem Formulation

### 3.1. Signal Generation and Receiver Chain

**Figure 1:**
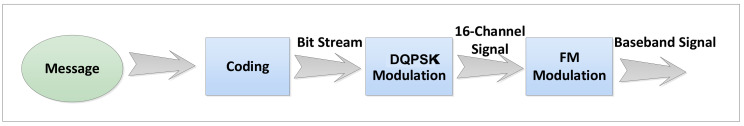
Signal generation process for the dual-modulated communication waveform.

The primary signal evaluated in this study is generated by a custom-developed practical data-link test system using a multi-tone DQPSK-FM composite waveform. The source message is encoded and mapped to differential QPSK (DQPSK) phase terms. These phase terms are then embedded in the multi-frequency FM-related payload expression used by the data-link waveform. The waveform is therefore described as a composite waveform throughout this paper. Figure 1 shows the signal-generation chain.

Let *m* denote the source message and let C(·) be the channel-coding operation. A simplified baseband description is

(1) b=C(m),

(2) $$\theta_{n}=P\left(b_{n}\right),$$

where b is the encoded bit stream and P(·) maps encoded bits to DQPSK phase increments. For the payload segment of the burst, the multi-frequency modulation signal can be written as

(3) $$u\left(t\right)=\sum_{i=1}^{16}k_{i}\cos\left(2\pi f_{i}t+\varphi_{i}+\theta_{i}\right),$$

where $f_{i}$ is the *i*th frequency component, $k_{i}$ is its amplitude coefficient, $\varphi_{i}$ is the carrier phase, and $\theta_{i}$ is the corresponding DQPSK phase term. The resulting complex baseband signal is represented as

(4) $$y\left(t\right)=A\exp\left(j\left(2\pi f_{c}t+k_{f}\int_{0}^{t}u\left(\tau \right)\;d\tau \right)\right),$$

where *A* is a normalization factor, $f_{c}$ is the carrier-related frequency term after baseband conversion, and $k_{f}$ is the FM modulation index. The multi-frequency expression above describes the payload segment. The synchronization preamble is handled separately. The preamble employs two synchronization frequencies (605 Hz and 2915 Hz), while the payload occupies 16 frequency components. To match the experimental signal format, the reference generator configures each frame with 30 encoded bits. These bits form 15 DQPSK symbol updates per frame, with phase increments selected from {±π/4,±3π/4}. The payload tone set is {935,1045,…,2365,2915,605} Hz. The FM stage uses $k_{f}=2\pi \times 6\times 3100$ after the modulation signal is amplitude-normalized.

The FSK generalization signal is a binary FSK waveform. In its native waveform generator, the complex phase is advanced by a positive or negative frequency state according to the current bit. The frequency separation parameter is set through a phase step of $2\pi \times 20\;\mathrm{kHz}/F_{s}$. The control burst uses a 16-bit alternating synchronization pattern at 10 kbit/s and a 5 kbit/s information section. The net-control and reply formats have different information lengths, so this class tests the denoiser on a simpler frequency-state modulation with different burst structures.

The frequency-hopping MSK generalization signal is generated from a time-slotted, pulse-based waveform. The native generator uses a 5 Mbps baseband rate and a 500 MHz sampling rate. One epoch contains 1536 time slots over 12 s, and each time slot has a duration of 7.8125 ms. Frequency hopping is enabled. The hop table contains 444 frequency entries per time slot, and pulse times are spaced by 13 μs. Each pulse carries a 35-bit MSK-modulated codeword before the receiver decoding stages. This dataset tests the denoiser on a time-varying spectral structure with continuous-phase modulation. Frequency-hopping waveform analysis is also important in emitter-identification tasks, where preserving fine waveform details supports robust feature extraction [25].

All signal classes use two-channel I/Q acquisition and the same segmentation protocol after receiver-side acquisition and slicing. The sampling rate used for the denoising dataset is 33 kHz. Each I/Q sequence contains 8192 samples. A demodulation frame contains 440 sampling points, so each sequence contains up to 18 complete frames and up to 540 bits. For every combination of signal class, disturbance or channel condition, and SNR point, 40,000 sequences were generated and split at the sequence level into 32,000 training sequences, 4000 validation sequences, and 4000 test sequences. Table 1 summarizes the common segmentation protocol, and Table 2 summarizes the signal-class settings.

At the receiver, the signal is downconverted, filtered, downsampled, synchronized, and demodulated. After initialization, the demodulator performs FM demodulation and filtering, detects the signal, completes frame synchronization, extracts the reference phase, acquires frame information, detects the signal end, and outputs the bit stream, constellation, and message type. This preamble-assisted procedure is used consistently in all BER/RBER comparisons.

### 3.2. Disturbance, Channel-Effect Model, and SNR Control

The disturbance and channel-effect cases were introduced by a channel simulator under controlled parameter settings. The channel simulator was connected to the same receiver chain used to acquire the interference-free waveform, so the paired samples share the same receiver processing path. Additive disturbance cases are expressed as

(5) r(t)=y(t)+n(t),

where r(t) is the received I/Q waveform, y(t) denotes the interference-free waveform, and n(t) is the additive disturbance. Multipath and Rayleigh cases are treated as channel effects. For these scenarios, the received waveform is modeled as

(6) r(t)=H{y(t)},

where H{·} denotes the channel operator. This explicitly distinguishes fading effects from additive noise.

The input SNR is the receiver-side power ratio between the target waveform and the disturbance component. For additive cases,

(7) $${\mathrm{SNR}}_{\mathrm{in}}=10\log_{10}\left(\frac{P_{y}}{P_{n}}\right),$$

where, for an $N_{s}$-point sampled I/Q sequence,

(8) $$P_{y}=\frac{1}{N_{s}}\sum_{q=1}^{N_{s}}|y_{q}{|}^{2},\;P_{n}=\frac{1}{N_{s}}\sum_{q=1}^{N_{s}}{\left|n_{q}\right|}^{2}.$$

For channel-effect cases, the same receiver-side SNR convention is applied to the power of the channel-induced residual d(t)=r(t)−y(t) after temporal alignment. Thus, all SNR values are measured at the receiver input and use the same sequence-level power calculation.

For an additive disturbance produced by the channel simulator, the unscaled disturbance component is denoted by $n_{0}\left(t\right)$, and target-SNR scaling is performed by

(9) $$n\left(t\right)=\alpha n_{0}\left(t\right),\;\alpha =\sqrt{\frac{P_{y}}{P_{n_{0}}10^{{\mathrm{SNR}}_{\mathrm{in}}/10}}},$$

where $P_{n_{0}}$ is the power of the unscaled disturbance. The channel simulator applies the specified disturbance or channel-effect parameters, and the receiver-side power setting is adjusted to the target SNR. This procedure keeps the signal source and receiver chain fixed and varies only the disturbance or channel-effect intensity.

**Figure 2:**
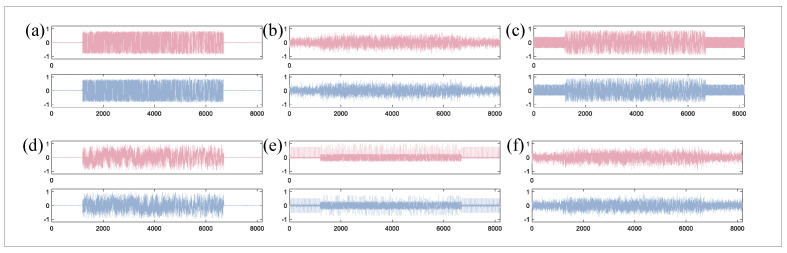
Received signals under selected disturbance and channel-effect conditions. (**a**) Interference-free signal; (**b**) AWGN; (**c**) frequency-modulated interference; (**d**) multipath fading; (**e**) comb-spectrum interference; (**f**) Rayleigh fading. The red and blue traces denote the I and Q components, respectively.

The channel-simulator conditions include AWGN, FM interference, wideband interference, AM interference, multipath fading, comb-spectrum interference, and Rayleigh fading. Representative examples of selected conditions are shown in Figure 2. Figure 2a gives the interference-free I/Q waveform used as the reconstruction target. The remaining panels illustrate typical waveform changes caused by the tested disturbances and channel effects.

Table 3 summarizes the channel-simulator source and generation parameters. AWGN is produced by the channel simulator as complex white Gaussian noise and normalized to zero mean and unit standard deviation before SNR scaling. The FM interference is produced by the channel simulator with center frequency $f_{J}=0$ Hz, frequency deviation Δf=10 kHz, and sinusoidal modulating signal q(t)=sin(2π·0.7kHz·t). The corresponding expression is

(10) $$n_{\mathrm{FM}}\left(t\right)=A_{n}\cos\left(2\pi f_{J}t+\Delta f\int_{0}^{t}q\left(\tau \right)\;d\tau \right),$$

where $A_{n}$ is the amplitude after target-SNR scaling. The wideband interference is produced in the channel simulator by passing complex white noise through an eighth-order low-pass IIR filter with a half-power frequency of 16.5 kHz. The AM interference is produced by the channel simulator with a carrier frequency of 50 Hz, a modulation frequency of 50 Hz, and a modulation index of 0.5.

Multipath fading is implemented in the channel simulator as a three-path channel effect,

(11) $${\tilde{r}}_{\mathrm{MP}}\left(t\right)=\sum_{\ell =1}^{3}c_{\ell }y\left(t-\tau_{\ell }\right),\;c_{l}=a_{\ell }e^{j\phi_{\ell }},$$

where the delay $\tau_{\ell }$ is drawn randomly from 0 to 1 ms and implemented by linear fractional-delay interpolation. With the sampling rate of 33 kHz, this delay range corresponds to approximately 0 to 33 sampling intervals, while the delay itself is not restricted to integer samples. The path amplitude $a_{\ell }$ is drawn randomly from 0.3 to 1, and the phase $\phi_{\ell }$ is drawn randomly from 0 to 2π. After the three delayed paths are summed, the multipath waveform is power-normalized before receiver-side SNR control,

(12) $$r_{\mathrm{MP}}\left(t\right)=\sqrt{\frac{P_{y}}{P_{{\tilde{r}}_{\mathrm{MP}}}}}\;{\tilde{r}}_{\mathrm{MP}}\left(t\right),$$

where $P_{{\tilde{r}}_{\mathrm{MP}}}$ is the average power of ${\tilde{r}}_{\mathrm{MP}}\left(t\right)$ computed over the I/Q sequence. Comb-spectrum interference is produced by the channel simulator and represented in the frequency domain as

(13) $$N_{\mathrm{comb}}\left(f\right)=\sum_{p=-7}^{7}\delta \left(f-pf_{0}\right),\;f_{0}=1000\;\mathrm{Hz},$$

which corresponds to 15 spectral lines with 1000 Hz spacing and unit amplitude before SNR scaling. Rayleigh fading is produced by the channel simulator as block flat multiplicative fading. For each 8192-sample I/Q sequence, one complex fading coefficient is generated as

(14) $$h=\frac{h_{I}+jh_{Q}}{\sqrt{2}},\;h_{I},h_{Q}∼N\left(0,1\right),$$

and is kept fixed over the whole sequence. The corresponding faded waveform is

(15) $$r_{\mathrm{Rayleigh}}\left(t\right)=hy\left(t\right).$$

This setting gives $E\{|h{|}^{2}\}=1$ and represents block flat Rayleigh fading without within-sequence Doppler variation.

### 3.3. Demodulation-Oriented Metric

**Figure 3:**
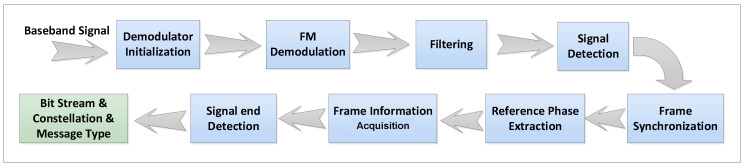
Workflow of the demodulator used for receiver-level evaluation.

**Figure 4:**
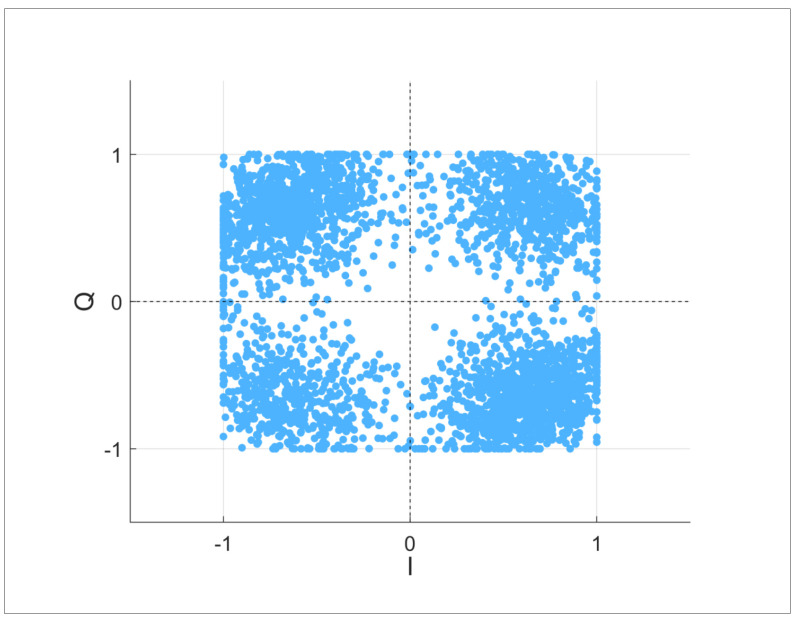
Representative demodulated constellation diagram.

Figure 3 summarizes the demodulator workflow, and Figure 4 shows a representative demodulated constellation. Because the final communication objective is correct bit recovery, waveform metrics are reported together with receiver-level BER. RBER is used to describe the residual BER penalty after denoising within the receiver transition region.

(16) $$\mathrm{RBER}=\frac{\mathrm{BER}\left(\hat{y}\right)-\mathrm{BER}\left(y\right)}{\mathrm{BER}\left(r\right)-\mathrm{BER}\left(y\right)+\epsilon_{\mathrm{BER}}},$$

where $\hat{y}=F_{\Theta }\left(r\right)$ is the denoised output of the neural network, BER(y) is the BER obtained from the interference-free waveform, and BER(r) is the BER of the corrupted received signal. The constant is fixed as $\epsilon_{\mathrm{BER}}=10^{-8}$ in all experiments and prevents division by zero. RBER is interpreted within the receiver transition region, where BER(r)−BER(y) is larger than the numerical floor set by $\epsilon_{\mathrm{BER}}$. When the denominator is dominated by $\epsilon_{\mathrm{BER}}$, the receiver is already in the high-SNR error-free region; therefore, BER and output SNR are reported directly and RBER is not used as the main comparison criterion.

## 4. Neural Denoising for Receiver Preprocessing

### 4.1. Learning Objective

The denoiser is used as a preprocessing module between the baseband receiver front end and the demodulator. It learns a mapping from a corrupted I/Q sequence to the corresponding interference-free sequence. For a training set ${\left\{\left(r_{i},y_{i}\right)\right\}}_{i=1}^{N}$, the objective is

(17) $$\Theta^{★}=\arg\min_{\Theta }\frac{1}{N}\sum_{i=1}^{N}{∥F_{\Theta }\left(r_{i}\right)-y_{i}∥}_{2}^{2},$$

where $F_{\Theta }\left(\;\cdot \;\right)$ denotes the denoising network. While RBER serves as the ultimate receiver-level metric, the network is optimized using MSE. This design choice bypasses the non-differentiable nature of synchronization, frame-boundary detection, and hard symbol-decision operations in conventional demodulators, preserving the denoiser as an independent, plug-and-play front end. Designing a BER-aware differentiable loss is left for future work. Each input sample contains two channels corresponding to the in-phase and quadrature components. Before training, each sequence is normalized as

(18) $$\tilde{r}=\frac{r-\mu_{r}}{\sigma_{r}+\epsilon_{\mathrm{std}}},$$

where $\mu_{r}$ and $\sigma_{r}$ are the sample mean and standard deviation, and $\epsilon_{\mathrm{std}}=10^{-8}$ is a small numerical constant that stabilizes the normalization denominator.

### 4.2. Network Implementation

A one-dimensional residual denoising autoencoder (DAE) is used as the receiver preprocessing module. The network is selected for nonlinear waveform restoration on long I/Q sequences and remains compatible with a conventional demodulation chain.

The implementation uses five encoder residual blocks, five decoder residual blocks, and a final convolutional mapping layer. Pooling is avoided because sample-level timing and phase information can be important for synchronization and symbol decisions. Full self-attention is also avoided in the proposed receiver front end because an 8192-point sequence would require a large attention map for each head. The key receiver features in this task are local phase continuity, preamble structure, and symbol-boundary preservation. Downsampling is performed by strided convolution, and upsampling is performed by transposed convolution. Batch normalization stabilizes feature scaling [26], parametric rectified linear unit (PReLU) activation supports negative-region gradients [27], and residual connections follow the standard residual-learning design [28]. The encoder and decoder kernel sizes are set to 23 and 5, respectively, based on the configuration sensitivity reported in Section 6.3. This configuration is fixed for all comparisons unless otherwise stated.

## 5. Experimental Setup

### 5.1. Datasets and Training Protocol

During data collection, the custom-developed signal generator, channel simulator, and receiver configuration were kept fixed except for the controlled disturbance or channel-effect strength. The input SNR was calculated from the received target-signal power and the received disturbance power according to Equations (7) and (8). Received samples were acquired under SNR values from −10 dB to 20 dB in 1 dB increments unless a narrower range was specified for a particular comparison. For each signal class, disturbance/channel condition, and SNR point, 40,000 I/Q sequences were generated. The dataset was randomly shuffled at the sequence level and split into 32,000 training sequences, 4000 validation sequences, and 4000 test sequences.

All neural models were trained for 200 epochs with a mini-batch size of 64. The optimizer was Adam with $\beta_{1}=0.9$, $\beta_{2}=0.999$, and a weight decay of $5\times 10^{-5}$. The initial learning rate was 0.002. A StepLR schedule was used with a step size of 8 epochs and a decay factor γ=0.2. Five repeated runs used random seeds 41, 42, 43, 44, and 45. The same split strategy, optimizer setting, learning-rate schedule, and seed protocol were used for the proposed model and neural baselines.

**Figure 5:**
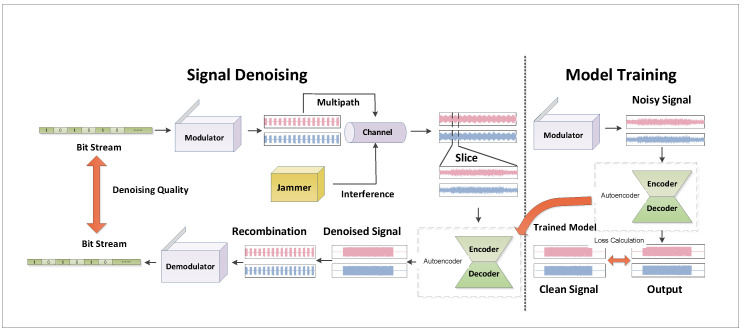
System framework for receiver-side neural denoising and demodulation-oriented evaluation. The left part illustrates the receiver-side denoising and BER-evaluation path; the right part illustrates paired model training with corrupted and interference-free waveforms. Only a short segment of the bit stream is shown for illustration.

Figure 5 summarizes the engineering evaluation framework. The left part shows the receiver-side denoising loop: a transmitted bit stream is modulated, corrupted by channel effects and interference, sliced into long I/Q samples, denoised by the trained front end, and then demodulated to evaluate bit recovery. The right part shows the supervised training loop, where paired corrupted and interference-free waveforms are obtained through the same signal source, channel simulator, and receiver chain and then used to optimize the autoencoder. The model is therefore assessed through the downstream demodulator and the resulting BER/RBER.

All neural networks and demodulator modules were implemented using Python 3.12, PyTorch 2.1.1+cu118, and CUDA 11.8. Training was performed on a workstation equipped with an Intel Core i9-13900HX central processing unit (CPU) and an NVIDIA RTX 6000 graphics processing unit (GPU). Each controlled experiment was repeated five times using random seeds 41–45 with the same channel-simulator protocol, and the reported curves or bars use the averaged result; error bars indicate run-to-run variation where shown. All model comparisons used identical data splits and training settings. The denoised waveform was passed to the same demodulator used for the corrupted and interference-free references; therefore, the reported BER change is attributable to the receiver preprocessing stage under a fixed demodulation chain.

### 5.2. Evaluation Metrics

The waveform-level error is measured by MSE.

(19) $$\mathrm{MSE}=\frac{1}{N_{s}}\sum_{q=1}^{N_{s}}{\left|{\hat{y}}_{q}-y_{q}\right|}^{2},$$

where $N_{s}=8192$ is the sequence length. The output SNR is calculated as

(20) $${\mathrm{SNR}}_{\mathrm{out}}=10\log_{10}\left(\frac{\sum_{q=1}^{N_{s}}{\left|y_{q}\right|}^{2}}{\sum_{q=1}^{N_{s}}{\left|{\hat{y}}_{q}-y_{q}\right|}^{2}}\right).$$

Because $N_{s}$ is fixed in all controlled comparisons, MSE and output SNR provide repeatable waveform diagnostics. They are not used as the sole evidence of receiver improvement. RBER in Equation (16) and the corresponding BER values are used to evaluate whether waveform reconstruction improves the actual demodulator output.

### 5.3. Baselines

Four representative denoising baselines are compared with the receiver DAE under matched data splits and training settings. The generative adversarial network (GAN) baseline uses a generator with the same main encoder–decoder structure and a discriminator with an encoder-like structure, following adversarial learning [29] and prior signal-restoration use [30]. The attention DAE inserts a self-attention module between the encoder and decoder [12,31]. The convolutional DAE (CDAE) removes residual skip paths from the proposed structure [32]. The pooling residual autoencoder (Res-AE) keeps residual blocks and replaces strided-convolution downsampling with pooling, following one-dimensional residual autoencoder practice for sequence signals [8]. These baselines isolate adversarial loss, global attention, residual skip paths, and pooling-based downsampling under the same demodulation-oriented evaluation protocol. Low-complexity filters remain useful when interference is spectrally separable. The present comparison focuses on neural front ends for overlapping disturbances that affect phase continuity and symbol decisions.

## 6. Results and Discussion

### 6.1. Model Comparison Under AWGN

All baseline models were trained under AWGN with an SNR of −1 dB and then evaluated from −5 dB to 1 dB. Table 4 reports the parameter count, MSE, and RBER. The receiver DAE gives the lowest RBER from −5 dB to −1 dB. At 0 dB and 1 dB, several neural models reach zero RBER after rounding, so these high-SNR points are not used to claim a unique best method. The attention model is competitive at high SNR, with weaker low-SNR RBER than the receiver DAE. The GAN baseline performs poorly in this receiver-oriented setting, indicating that adversarial restoration can introduce waveform artifacts that are penalized by the demodulator. The pooling residual autoencoder shows that pooling-based downsampling can remove details that remain relevant to synchronization and symbol decisions.

### 6.2. Inference Complexity and Deployment Scope

The receiver DAE contains approximately 61.61 million trainable parameters. Using uncompressed 32-bit floating-point precision, parameter storage requires approximately 246 MB. Quantization to 16-bit floating-point or 8-bit integers can reduce this to 123 MB or 62 MB, respectively, excluding activation buffers and implementation overhead. Therefore, the current implementation is best interpreted as an offline performance upper-bound and receiver-chain feasibility study. For practical deployment in field-programmable gate array (FPGA), embedded GPU, or digital-signal-processing hardware, the model would require latency profiling together with pruning, quantization, channel reduction, streaming convolution, or knowledge distillation.

### 6.3. Configuration Sensitivity

**Figure 6:**
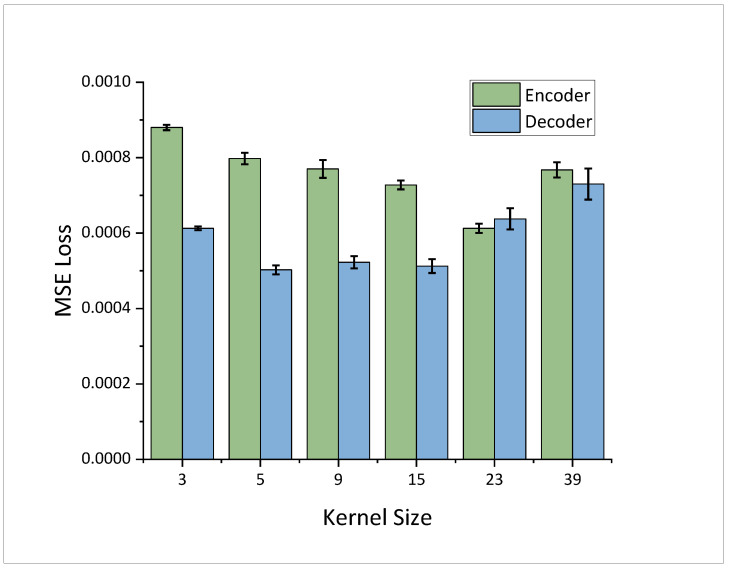
Configuration sensitivity to encoder and decoder convolution kernel sizes.

The convolution kernel size was varied to select a stable receiver-front-end configuration. Figure 6 compares encoder and decoder kernel settings using MSE loss. The green bars show that the encoder benefits from a larger kernel because a wider receptive field captures the local waveform context around preambles, symbol transitions, and FM-induced phase evolution. The best encoder result occurs at a kernel size of 23. The blue bars show that the decoder does not benefit from continuously increasing its kernel; after a moderate value, the additional smoothing can slightly degrade reconstruction. The final model therefore uses an encoder kernel size of 23 and a decoder kernel size of 5, and this configuration is then assessed through BER/RBER in the following experiments.

### 6.4. Training-SNR Sensitivity

**Figure 7:**
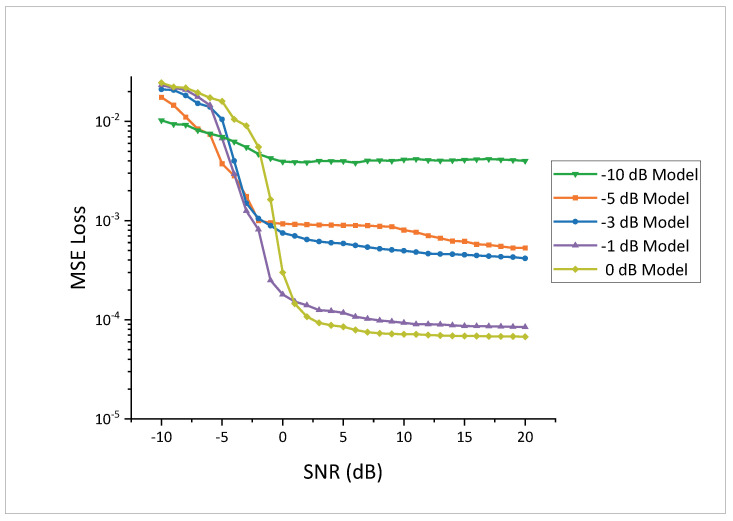
MSE performance of models trained under different SNR conditions. The vertical axis uses a logarithmic scale.

To evaluate sensitivity to training SNR, separate models were trained under −10 dB, −5 dB, −3 dB, −1 dB, and 0 dB AWGN and then tested from −10 dB to 20 dB. Figure 7 shows that models trained at −5 dB, −3 dB, and −1 dB generalize well once the test SNR is above approximately −2 dB. The −10 dB model remains more tolerant in the very-low-SNR region but leaves a higher residual error floor after the input SNR improves. The 0 dB model converges quickly at moderate SNRs but is less reliable in the harshest range. This pattern supports choosing the training SNR near the receiver transition region. The easiest condition alone is insufficient.

**Figure 8:**
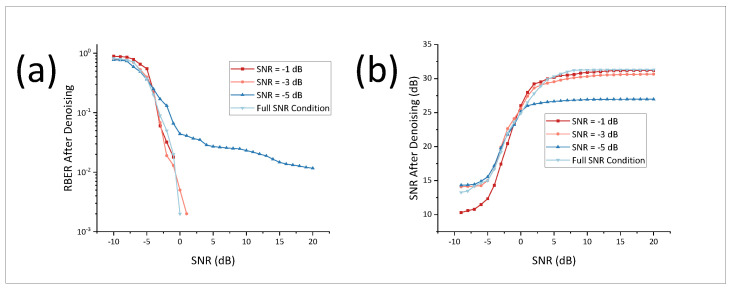
Comparison between single-SNR training and the Full SNR Condition model. (**a**) RBER after denoising on a logarithmic vertical axis; (**b**) output SNR after denoising.

A model labeled Full SNR Condition in Figure 8 was trained using samples from the five training-SNR conditions. Figure 8a compares RBER after denoising. The −1 dB and −3 dB single-SNR models reduce the BER penalty rapidly as the test SNR approaches the receiver operating region. The −5 dB model keeps a small residual RBER over a wider high-SNR interval. Figure 8b shows the corresponding output SNR after denoising. The Full SNR Condition model provides stable average behavior and a high output-SNR ceiling. A carefully selected single-SNR model can still be stronger in part of the receiver operating region. These results show that a moderately difficult SNR can be an effective training target when exhaustive SNR mixing is not available for every receiver condition.

### 6.5. Generalization Across Disturbance Conditions and Signal Types

**Figure 9:**
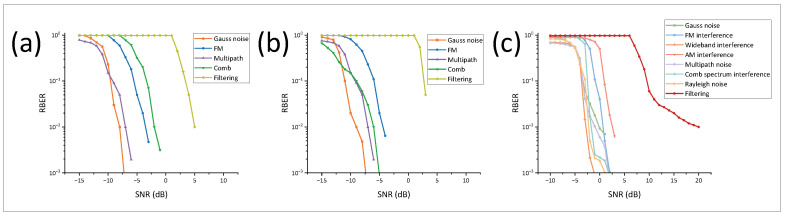
Generalization under different disturbance/channel-effect conditions and signal types. All panels use logarithmic RBER axes. (**a**) RBER on the binary FSK dataset; (**b**) RBER on the frequency-hopping MSK dataset; (**c**) RBER of the main multi-tone DQPSK-FM composite waveform under the selected disturbance and channel-effect conditions, with filtering included as a receiver reference.

Figure 9 adds the disturbance-condition and signal-type tests using logarithmic RBER axes. Figure 9a,b test the additional binary FSK and frequency-hopping MSK datasets described in Table 2. In the FSK case, the RBER transition occurs at lower SNRs under AWGN than under FM interference, multipath fading, comb-spectrum interference, or the filtering reference. The frequency-hopping MSK case shows the same general trend, but FM interference and comb-spectrum interference shift the transition to higher SNR because the waveform contains hop-dependent spectral movement and short pulse structures.

Figure 9c tests the main multi-tone DQPSK-FM composite waveform under the selected disturbance and channel-effect conditions. The DAE used for this comparison was trained at −1 dB under AWGN and then evaluated under AWGN, FM interference, wideband interference, AM interference, multipath fading, comb-spectrum interference, and block flat Rayleigh fading. For most neural-denoising cases, the residual BER penalty falls toward the plotting floor between approximately −4 dB and 2 dB, depending on the tested condition. The filtering reference requires a much higher SNR before the BER penalty is reduced. These results indicate that the receiver front end extends beyond the main waveform, while the exact operating threshold still depends on the modulation format and disturbance or channel-effect structure.

## 7. Conclusions

This study presented demodulation-oriented neural denoising for long-sequence I/Q wireless signals in a data-link receiver chain. The denoiser is inserted before demodulation and evaluated by MSE, output SNR, BER, and RBER, linking waveform restoration to receiver-level bit recovery.

Experiments on three communication-signal datasets and representative receiver-side disturbance conditions show that the neural denoising stage improves low-SNR demodulation accuracy compared with convolutional, pooling-based, attention-based, and adversarial baselines. The training-SNR experiments show that the training distribution should cover the receiver transition region, where small waveform changes can produce large BER changes. The added disturbance-condition and signal-type tests further show that the method generalizes beyond the main dual-modulated waveform, while the remaining BER penalty depends on the disturbance and channel-effect structure. Future work will focus on lower-complexity receiver front ends, pruning and quantization for real-time implementation, unsupervised or weakly supervised adaptation for changing electromagnetic environments, public benchmarks for long I/Q denoising, and joint denoising–demodulation models that optimize waveform reconstruction and bit recovery within one receiver chain.

## Figures and Tables

**Table 1:** I/Q segmentation and dataset split settings used for all signal classes.

Item	Value
Sampling rate	33 kHz
I/Q sequence length	8192 samples
Sampling points per frame	440 samples
Maximum number of frames per sequence	18
Maximum number of bits per sequence	540 bits
Total sequences per signal class/condition/SNR point	40,000 sequences
Training/validation/test sequences per combination	32,000/4000/4000 sequences
Split level	Random sequence-level split

**Table 2:** Main modulation and burst-structure settings of the three signal classes. Native generator rates identify the source waveform before receiver-side acquisition, slicing, or resampling to the common denoising dataset format.

Signal Class	Native Generator Settings	Modulation and Frame Structure Used in the Experiments
Multi-tone DQPSK-FM composite waveform	$F_{s}$ is constrained to an integer multiple of 75 Hz; the denoising dataset uses 33 kHz after receiver-side acquisition.	A frame contains 30 encoded bits and 15 DQPSK phase updates. The phase increments are ±π/4 and ±3π/4. The payload uses 16 frequency components, 935:110:2365 Hz plus 2915 Hz and 605 Hz. The preamble uses 605 Hz and 2915 Hz. The FM stage uses $k_{f}=2\pi \times 6\times 3100$ after modulation-signal normalization.
Binary FSK waveform	Native reference generator: 200 kHz sampling rate; 5 kbit/s information section; 10 kbit/s synchronization section.	The complex baseband phase is advanced according to the bit-dependent frequency state. The phase-step parameter is $2\pi \times 20\;\mathrm{kHz}/F_{s}$, corresponding to the positive and negative FSK states in the native generator. The burst uses a 16-bit alternating synchronization pattern, a 5-bit start field, an information field, and an end bit. Net-control and reply bursts are both included in the reference format.
Frequency-hopping MSK waveform	Native reference generator: 5 Mbps baseband rate and 500 MHz sampling rate. One epoch contains 1536 slots over 12 s; each slot is 7.8125 ms.	Frequency hopping is enabled. The hop table contains 444 frequency entries per time slot. Pulse arrival times are spaced by 13 μs. Each pulse carries a 35-bit MSK-modulated codeword before decoding. The tested setting uses the STDP message type, one target, repetition-rate setting 15, slot jitter enabled, and source/channel scrambling enabled.

**Table 3:** Channel-simulator source and key parameters of the disturbance and channel-effect cases.

Condition	Type	Source	Key Parameters
AWGN	Additive disturbance	Channel simulator	Complex Gaussian white noise; normalized to zero mean and unit standard deviation before target-SNR scaling.
FM interference	Additive structured interference	Channel simulator	Center frequency 0 Hz; frequency deviation 10 kHz; modulating signal is a 0.7 kHz sinusoid.
Wideband interference	Additive filtered noise	Channel simulator	Complex white noise filtered by an eighth-order low-pass IIR filter; half-power frequency 16.5 kHz.
AM interference	Additive structured interference	Channel simulator	Carrier frequency 50 Hz; modulation frequency 50 Hz; modulation index 0.5.
Multipath fading	Channel effect	Channel simulator	Three paths; random 0–1 ms delays implemented by linear fractional-delay interpolation; random complex path coefficients with amplitudes from 0.3 to 1 and phases from 0 to 2π; power normalization after path summation.
Comb-spectrum interference	Additive structured interference	Channel simulator	Base frequency 1000 Hz; spacing 1000 Hz; 15 spectral lines; unit line amplitude before SNR scaling.
Rayleigh fading	Channel effect	Channel simulator	Block flat multiplicative fading; one complex coefficient per I/Q sequence, $h=\left(h_{I}+jh_{Q}\right)/\sqrt{2}$ with $h_{I},h_{Q}∼N\left(0,1\right)$.

**Table 4:** Performance comparison of denoising models under AWGN. Parameter counts are reported in millions.

Method	Params (M)	Metric	Input SNR (dB)						
			−5	−4	−3	−2	−1	0	1
GAN	94.85	MSE	0.0259	0.0250	0.0246	0.0169	0.0161	0.0079	0.0042
		RBER	0.9665	0.9652	0.9519	0.7164	0.6731	0.6002	0.0459
Attention DAE	63.07	MSE	0.0154	0.0082	0.0050	**0.0024**	0.0012	**0.0005**	**0.0003**
		RBER	0.6931	0.5914	0.0664	0.0536	0.0195	**0.0000**	**0.0000**
CDAE	61.61	MSE	0.0214	0.0174	0.0067	0.0047	0.0026	0.0023	0.0017
		RBER	0.8841	0.7878	0.5261	0.0697	0.0558	0.0467	0.0294
Pooling Res-AE	55.81	MSE	0.0152	0.0084	0.0057	0.0030	0.0014	0.0010	0.0005
		RBER	0.6815	0.6103	0.0696	0.0574	0.0203	0.0109	**0.0000**
Receiver DAE	61.61	MSE	**0.0146**	**0.0068**	**0.0046**	**0.0024**	**0.0011**	**0.0005**	0.0004
		RBER	**0.6530**	**0.5504**	**0.0600**	**0.0518**	**0.0151**	**0.0000**	**0.0000**

Note: Bold values indicate the best result for each metric at each input SNR; ties are shown in bold.

## Data Availability

The raw hardware-collected communication-signal records are available from the corresponding author upon reasonable request, subject to institutional restrictions. The synthetic interference-generation scripts and PyTorch implementation of the receiver DAE can also be made available upon reasonable request, subject to institutional approval.
